# Cystic neutrophilic granulomatous mastitis with corynebacterium and staphylococcus mimicking breast carcinoma

**DOI:** 10.1002/ccr3.1801

**Published:** 2018-10-10

**Authors:** Yihong Wang, Mark LeGolvan, Kimberle Chapin, Martha Mainiero

**Affiliations:** ^1^ Department of Pathology and Laboratory Medicine Rhode Island Hospital and Alpert Medical School of Brown University Providence Rhode Island; ^2^ Department of Diagnostic Imaging Rhode Island Hospital and Alpert Medical School of Brown University Providence Rhode Island

**Keywords:** breast carcinoma, corynebacterium, cystic neutrophilic granulomatous mastitis, staphylococcus

## Abstract

Cystic neutrophilic granulomatous mastitis (CNGM) is an increasingly recognized entity in the differential diagnosis of granulomatous inflammation involving the breast. We present the first case report of CNGM mimicking carcinoma of the breast with two mixed bacterial species as the causative pathogens (*Corynebacterium* and *Staphylococcus* spp.).

## 1 INTRODUCTION

We present a case of a 52‐year‐old woman with a tender mass in her left breast imaging studies suspicious for cancer. Biopsy revealed cystic neutrophilic granulomatous mastitis with characteristic cystic spaces lined by neutrophils with Gram‐positive cocci. 16S NGS identified both *Corynebacterium* and *Staphylococcus* spp.

In 2003, Taylor et al^1^ reported a landmark series of 34 breast abscesses with distinct histologic features that were associated with *Corynebacterium* infection. Most cases were granulomatous, but all cases had a suppurative component with distinct vacuoles that were thought to represent dissolved lipid. Organisms were identified by Gram stain in 16 cases, but in every case the organisms were rare, often missed on initial review of the Gram stain, and found only within the vacuoles. The appreciation of the distinct features of *Corynebacterium* infection in breast abscesses has not been well recognized until recently. Additional case series by Renshaw et al and D'Alfonso et al have defined this entity as cystic neutrophilic granulomatous mastitis (CNGM), with characteristic histology and a strong association with corynebacterium.^2^, ^3^ Clinically, CNGM manifests as a palpable mass of which half were tender. In two of nine cases reported by D'Alfonso, imaging was highly suspicious for malignancy (BI‐RADS 5). Histologically, they are characterized by variably well‐formed granulomas remarkable for cystic spaces lined by a cuff of neutrophils with rare Gram‐positive bacilli. Corynebacterium was identified within the cystic space in over half of the reported cases. We describe the clinicopathologic features of a case of breast CNGM clinically mimicking breast carcinoma and highlight the importance of recognizing this diagnosis.

## 2 CASE

**Figure 1:**
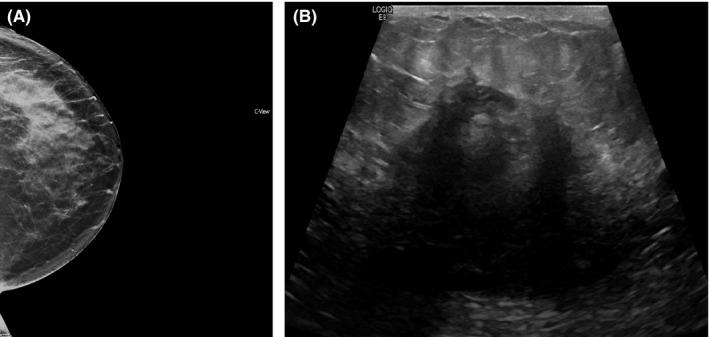
Left breast mammogram revealing asymmetry (A) and ultrasound showing an irregular mass with shadowing (B)

A 52‐year‐old woman with no significant past medical history presented with a 2‐week history of a mass in her left breast which was tender to palpation. A mammogram of the left breast revealed a large area of asymmetry laterally middle to posterior in‐depth new compared to her prior mammograms. On ultrasound, there was a corresponding irregular mass measuring approximately 4.5 cm suspicious for breast carcinoma, although mastitis was also considered (Figure 1).

**Figure 2:**
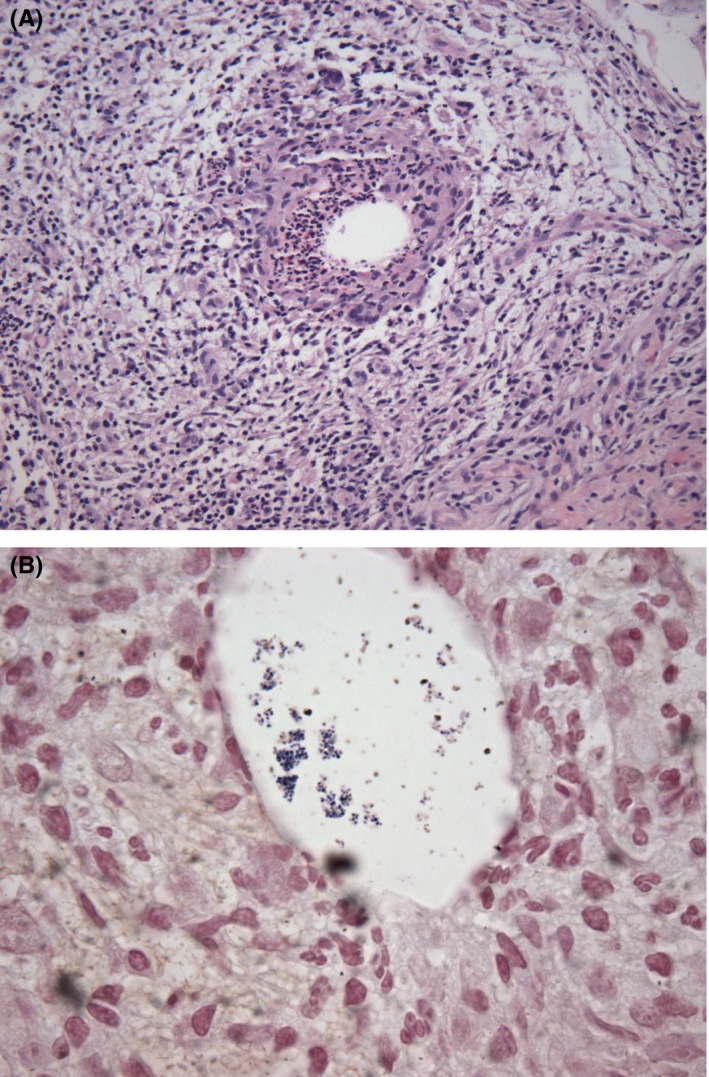
H&E section at 200× (A); Gram stain at 1000× (B)

An ultrasound‐guided needle biopsy was performed, and pathologic examination revealed an acute mastitis with a granulomatous component. At low power, a brisk neutrophilic inflammatory infiltrate was noted in the breast parenchyma which in some foci appeared to be centered to ducts and lobules. A subset of the granulomas was remarkable for cystic spaces of varying size (so‐called lipogranulomas). The cystic spaces were lined by a cuff of neutrophils, and Gram‐positive cocci were identified within and at the edge of the cystic spaces (Figure 2). Special stains including PAS‐D, GMS, and AFB were negative for fungal organism and acid‐fast bacilli. Because of the characteristic histology, corynebacterium was also suggested despite the identification of Gram‐positive cocci in the diagnostic comment. Subsequent 16S NGS identified *Corynebacterium bovis* (major abundance) and *Staphylococcus warneri* or *Staphylococcus pasteuri* (moderate abundance).

To our knowledge, this is the first reported case of CNGM mimicking carcinoma of the breast with a mixed bacterial population as the causative pathogens (Figure 2).

## 3 DISCUSSION

The differential diagnosis for granulomatous disease of the breast includes various infectious etiologies, reaction to exogenous material, and sarcoidosis. Idiopathic granulomatous mastitis might be rendered after excluding other etiologies.^4^, ^5^ Cystic neutrophilic granulomatous mastitis is a recently recognized entity should be included in the differential diagnosis of granulomatous inflammation involving the breast.

The clinical presentation and histologic findings in this case are the characteristics of CNGM. In brief, a young woman presented with the recent development of a tender mass in her breast. The imaging findings were worrisome for malignancy. Histologically, granulomatous inflammation with a lobulocentric distribution was present, and a subset of the granulomas contained cystic spaces. These cystic spaces were secondary to a central lipid vacuole and are analogous to the fibrin‐ring granuloma most commonly associated with Q‐fever (*Coxiella burnetti*) without the neutrophils.^6^ Gram‐positive cocci instead of the bacilli of coryneform bacteria were identified within the cystic spaces of some of the suppurative lipogranulomas. Due to the histologic characteristics and discordant bacterial form on Gram stain, 16S NGS was performed and both *C. bovis* and Staphylococci spp. were identified, consistent with both the Gram stain impression and the characteristic histologic findings. Interestingly, while *C. bovis* (a zoophile) has been recovered in a blood culture in humans, it has not yet been reported as one of the *Corynebacterium* sp. recovered from CNGM, whereas, in its host, the cow, its prime pathogenicity as a lipophile is in fact mastitis.^7^

In summary, we present an entity that is difficult to distinguish from breast carcinoma without pathologic examination. Corynebacterium should be considered when the constellation of histologic findings, such granulomatous inflammation with formation of suppurative lipogranulomas, is present even without the presence of an organism. Of note, *Corynebacterium* spp. tend to be scant in quantity and organisms may be difficult to culture. Careful histologic evaluation, especially in the cystic spaces, is critical for both single and mixed organism identification.

## CONFLICT OF INTEREST

None declared.

## AUTHORS CONTRIBUTIONS

YW: conceived and prepared the manuscript. ML, KC, and MM: helped prepare and revised the manuscript. All authors: read and approved of the final manuscript.
